# A mechanistic hypothesis: osteopathic manipulative therapy may modulate immune cell function in chronic low back pain

**DOI:** 10.3389/fpain.2026.1661536

**Published:** 2026-04-21

**Authors:** Lily Tehrani, Jackson Gamer, Sarah Ballarin, Sebastian Arango, Nathan Widboom, Patrick Barry, Mark Sandhouse, Jill Wallace-Ross, Yasmin Qureshi, Lubov Nathanson

**Affiliations:** 1 Kiran C. Patel College of Osteopathic Medicine, Nova Southeastern University, Fort Lauderdale, FL, United States; 2 Department of Pediatrics, Nationwide Children’s Hospital, Ohio State University College of Medicine, Columbus, OH, United States; 3 Department of Osteopathic Principles and Practice, Kiran C. Patel College of Osteopathic Medicine, Nova Southeastern University, Fort Lauderdale, FL, United States; 4 Institute for Neuro Immune Medicine, Kiran C. Patel College of Osteopathic Medicine, Nova Southeastern University, Fort Lauderdale, FL, United States

**Keywords:** chronic low back pain, immune system, lymphatic system, osteopathic manipulative therapy, transcriptomics

## Abstract

Chronic low back pain (CLBP) remains a leading cause of disability worldwide and is increasingly recognized as a condition that involves immune-mediated neuroinflammatory mechanisms. Osteopathic manipulative therapy (OMT) is a widely used non-invasive, non-pharmacological treatment for chronic pain; however, the molecular mechanisms underlying OMT effectiveness are not fully understood. We propose a mechanistic hypothesis of how OMT may influence circulating immune-cell gene expression and immune-cell distribution, with downstream effects on inflammatory signaling and neuroimmune sensitization. The mechanical forces applied during OMT could affect immune cell function, including leukocyte trafficking and cytokine activity. These mechanical influences may alter transcriptional activity within peripheral blood mononuclear cells, and over time, contribute to changes in gene expression patterns. This hypothesis article synthesizes current evidence from immunology, mechanobiology, and osteopathic clinical research to outline a proposed transcriptomic framework linking OMT to neuroimmune regulation in CLBP. This hypothesis supports a possible role for OMT in chronic pain management and suggests that immune modulation may represent one potential mechanism contributing to therapeutic benefit in persistent pain conditions. While current evidence suggests that OMT may influence immune function, direct transcriptomic validation remains limited and warrants further investigation through larger mechanistic studies.

## 1 Introduction

Chronic pain is a debilitating condition affecting over 500 million people globally that puts a significant strain on healthcare systems and contributes to poor quality of life (1). In the United States, chronic lower back pain (CLBP) is the leading cause of disability (2). Therapeutic management of CLBP varies widely, ranging from medications and stretching to surgical interventions (3). One significant challenge to the current treatment options is the reliance on long-acting non-steroidal anti-inflammatory drugs (NSAIDs) and opioids. While these potent analgesics can provide temporary relief, they are often accompanied by severe side effects. For example, opioids are associated with addiction and mortality from overdose, and chronic NSAID use increases the risk of peptic ulcer disease, acute renal failure, and stroke (4, 5). These limitations emphasize the need for effective, non-invasive, and non-pharmacological interventions to address CLBP.

**Figure 1:**
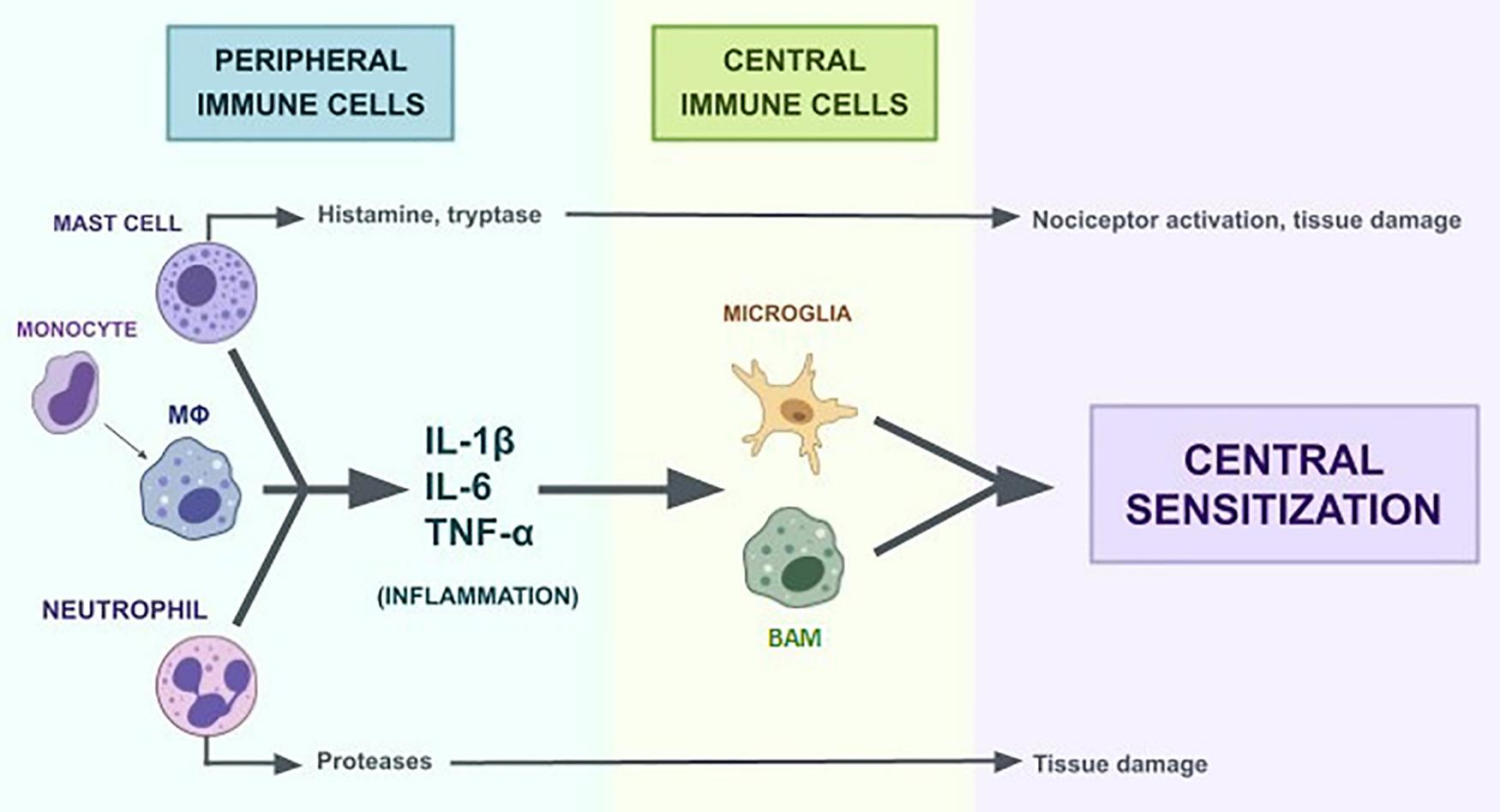
Overview of the interactions between peripheral and central immune cells involved in the pathophysiology of chronic low back pain. Mast cells, monocytes, macrophages (MΦ) and neutrophils are peripheral immune cells that, when activated by circulating cytokines or other inciting factors, produce a cascade of effects that contribute to inflammation (6). Activated monocytes differentiate into macrophages. Macrophages, mast cells, and neutrophils release various pro-inflammatory cytokines (IL-1β, IL-6, TNF-α, etc.) (119–121). Cytokines will travel to the dorsal horn of the spinal cord and the brain to interact with microglia and border-associated-macrophages (BAMs) (108). These central immune cells facilitate central sensitization through release of pro-inflammatory cytokines (110–113).

Both peripheral immune activation and central nervous system sensitization contribute to chronic low-back pain (Figure 1) (6–8). At the periphery, macrophages, mast cells, and T cells release cytokines that sensitize nociceptors and maintain inflammation. Within the central nervous system, microglia and astrocytes become activated and reinforce hyperexcitability in pain pathways through cytokine and neurotransmitter signaling (9). Effective therapies must therefore target both peripheral inflammatory mediators and central neural plasticity to achieve durable pain relief.

Recognizing this problem, the National Institutes of Health launched the Helping to End Addiction Long-term (HEAL) initiative in 2018 to advance strategies for chronic pain, including exploring promising approaches such as osteopathic manipulative therapy (OMT) (10). OMT utilizes hands-on techniques to target musculoskeletal, lymphatic, and neurovascular structures. Meta-analyses have demonstrated the efficacy of OMT in reducing pain and improving function in patients with CLBP (11–18). Despite its potential, the biological mechanisms underlying OMT's therapeutic effects remain poorly understood.

Recent advances in pain research have shown that the previously overlooked immune system is central to chronic pain conditions (19). Although the feeling of pain is processed in the nervous system, sustained inflammation contributes to the perpetuation of chronic pain and immune cells have been implicated in modulating the excitability of pain pathways (20). As part of this process, circulating immune cells undergo transcriptional changes that have been linked to the transition from acute to chronic pain or the resolution of nociceptive hypersensitivity (21). To date, multiple research efforts have been directed toward understanding how chronic pain develops; however, the biological mechanisms underlying pain management and resolution have remained largely unexplored. To address this gap, our hypothesis serves to bridge current existing findings by combining evidence of the efficacy of using OMT to reduce CLBP and the transcriptomic changes in circulating immune cells following OMT treatment. More specifically, our hypothesis is the first to postulate the mechanistic link between OMT, immune system modulation, and pain reduction.

**Figure 2:**
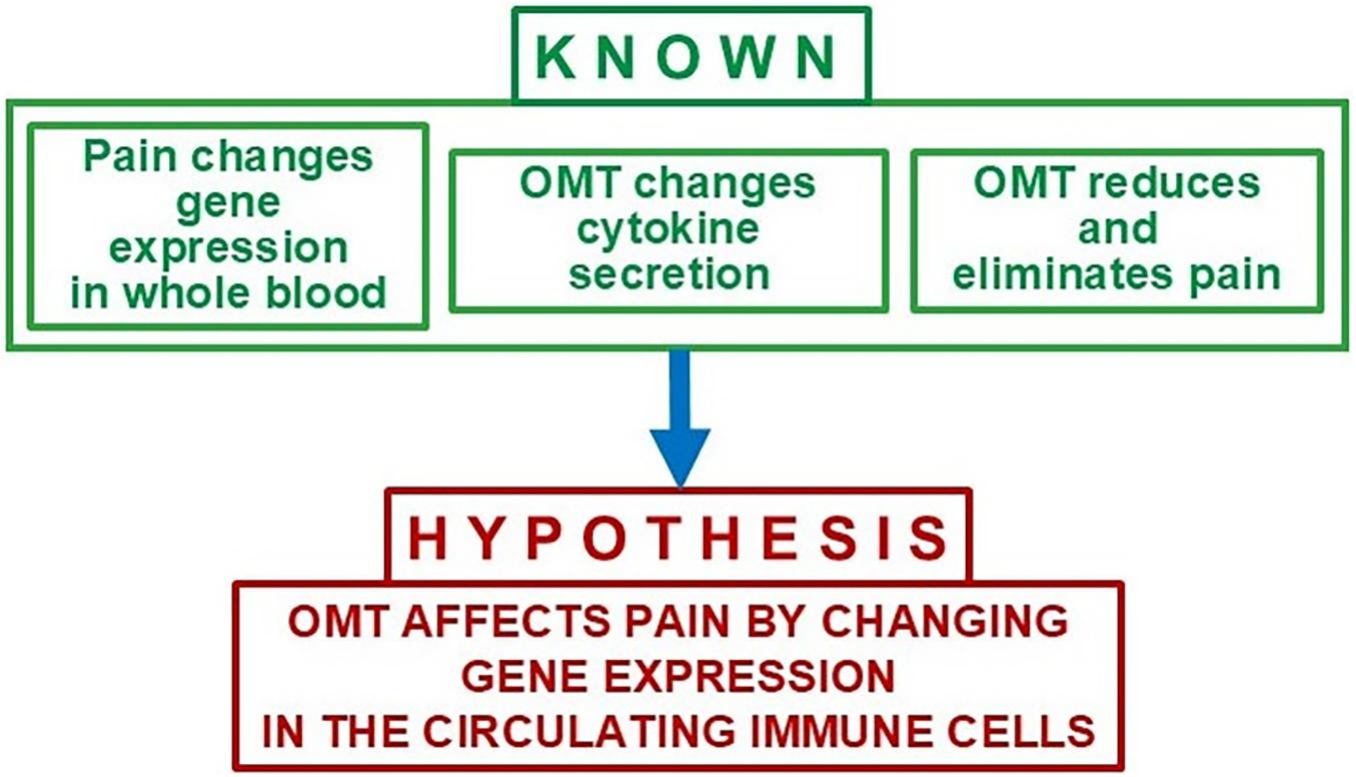
Rationale and central hypothesis for the mechanism of pain reduction by OMT.

This manuscript proposes a novel hypothesis: OMT alleviates pain in patients with CLBP by modulating immune cell activity. Specifically, the manipulation of soft tissue and bony structures exerts mechanical pressure on lymph nodes and lymphatic vessels that trigger shifts in immune cell proportions and activation states. These changes are mediated by transcriptomic alterations in circulating immune cells, involving genes associated with reduced cytokine production and the reprogramming of immune responses. These immune modulations influence central nervous system (CNS) pathways involved in pain and pain perception. (Figure 2).

A structured literature search was conducted using PubMed and Embase databases. Search terms included combinations of “osteopathic manipulative treatment,” “manual therapy,” “chronic low back pain,” “lymphatic pump,” “cytokines,” “immune modulation,” and “gene expression.” Both foundational osteopathic literature and more recent mechanistic studies were reviewed to capture evidence supporting and opposing immune changes following OMT and related manual therapies. Emphasis was placed on randomized controlled trials, translational models, and studies focusing on inflammatory mediators or transcriptomic outcomes.

## 2 Support for the hypothesis

### 2.1 The role of the immune system in chronic pain including CLBP

Chronic pain is increasingly recognized as a complex, multifactorial condition, with persistent inflammation playing a central role in its pathophysiology. Local immune cells, including macrophages, mast cells, and T cells, release a cascade of pro-inflammatory mediators, such as bradykinin, prostaglandins, and cytokines (e.g., TNF-α, IL-1β, IL-6), which sensitize peripheral nociceptors and amplify pain signaling pathways (6). Macrophages, in particular, play a dynamic role in pain pathways. When activated toward the M1 state, they release TNF-α and IL-1β that sustain inflammation and nociception, while the M2 phenotype helps resolve pain through tissue repair. T-cell subsets, such as Th17 and regulatory T cells, can also shift this balance by either increasing or calming pain signals depending on the cytokine environment (22). Cytokines like IL-6 and IL-10 can act in both directions, with their effects influenced by timing, concentration, and local context (6, 23, 24). It is important to note that cytokines are not always harmful and that their effects depend on timing and context. For example, IL-6, TNF-α, and IL-10 can drive inflammation when persistent but also play important roles in repair and immune balance once the inflammatory phase resolves. This balance between injury and recovery is what OMT may help recreate, shifting cytokine activity toward resolution rather than a pathological role (25).

Tissue-specific macrophages within the musculature, vasculature, and nervous system, along with monocyte-derived macrophages, play a dynamic role in the pathophysiology of CLBP (26–28). When exposed to pathogen-associated molecular patterns (PAMPs) or damage-associated molecular patterns (DAMPs), macrophages polarize toward the proinflammatory M1 phenotype and secrete cytokines such as TNF-α and IL-1β, which sustain location inflammation and sensitive nociceptive pathways (29–32). Macrophages also actively engulf damaged cells and secrete lysosomal enzymes that contribute to the intercellular breakdown of substances in these tissues (33–36). As tissue injury resolves, macrophages undergo a phenotypic shift toward the anti-inflammatory M2 state, characterized by the release of mediators that promote tissue remodeling, angiogenesis, and wound repair (37–41). This transition from M1- to M2-dominant activity is essential for the resolution of inflammation and attenuation of pain (22, 42, 43) and may be disrupted in patients with CLBP. For example, studies of chronic lumbar radiculopathy and intervertebral disc degeneration have demonstrated a predominance of M1 macrophage-mediated inflammation (27, 44).

These inflammatory mediators activate intracellular signaling cascades, including protein kinase C and mitogen-activated protein kinases, further lowering the activation threshold of nociceptors (45). Sustained inflammation at these nerve endings promotes the release of excitatory neurotransmitters such as glutamate, substance P, and calcitonin gene-related peptide in the dorsal horn of the spinal cord (7, 46–49). This cascade of events contributes to the phenomenon of central sensitization, characterized by heightened neuronal excitability, expanded receptive fields, and diminished inhibitory modulation of pain signals (Figure 1) (8). Specifically, in CLBP neuroimaging and biomarker studies have revealed distinct patterns of neuroinflammation and maladaptive neuroplasticity, which vary depending on clinical presentation (50–53). Peripheral immune activation interacts with central mechanisms through cytokine-mediated glial activation, linking local inflammation to neuroimmune sensitization in the spinal cord and brain (7, 8, 54–57).

### 2.2 Chronic pain including CLBP induces gene expression changes in immune cells

Research has shown distinct changes in the gene expression of immune cells as a result of chronic pain conditions such as CLBP. For example, a study published in 2019 identified a unique CLBP-specific transcriptome in whole blood, notably enriched for genes involved in the antigen presentation pathways (58). Additional research has revealed thousands of dynamic transcriptional changes in circulating immune cells in CLBP patients, distinguishing those who resolved their pain from those with persistent symptoms (59). Beyond transcriptional changes, recent studies have uncovered epigenetic modifications in immune cells that uniquely characterize CLBP and exhibit sex-specific differences. For instance, DNA methylation signatures in T cells have been identified as unique to CLBP patients, enabling reliable differentiation from healthy controls (HCs) (60). These findings highlight the role of transcriptional and epigenetic reprogramming in circulating immune cells in the pathophysiology of CLBP.

Despite growing evidence that chronic pain alters immune system, studies evaluating whether manual therapies can reliably modify circulating inflammatory markers have yielded mixed results. For example, Degenhardt and Johnson reported no significant differences in IL-1β, IL-6, TNF-α, or CRP following manual treatment in individuals with CLBP, despite improvements in self-reported pain (61). A narrative overview of manual therapy and cytokine research described significant variability across studies, noting that immune responses appear to depend on timing of measurement, baseline inflammatory states, and treatment parameters (62). Together, these findings suggest that immune modulation following manual therapies is variable, and these inconsistencies highlight the need for future studies to clarify whether transcriptomic profiling may provide a more sensitive measure of immune modulation than single time-point cytokine assays.

### 2.3 Clinical evidence of OMT efficacy in reducing CLBP

CLBP can be classified based on the source of pain, though these often overlap in clinical practice**.** For instance, discogenic pain arises from degenerative changes within the intervertebral discs that trigger cytokine release, nerve growth, and inflammation (63, 64). Facetogenic pain is more mechanical, caused by irritation or arthritis in the facet joints (65–67). Radicular pain develops when nerve roots are compressed or inflamed (68), while myofascial pain arises from tight or irritated muscle and fascia that can form trigger points (69, 70). Sacroiliac pain and mixed patterns add another layer of complexity, since many patients have more than one source of pain at the same time (71, 72). Although these processes begin differently, they often share downstream pathways involving neuroinflammation and central sensitization (73, 74). Most patients presenting with CLBP are classified as having nonspecific pain, meaning that their symptoms occur in the absence of an identifiable, specific underlying pathology (75). Fewer than 10 percent of patients have pain attributable to a specific subacute or chronic etiology. In these cases, pain often arises from degenerative changes within the vertebral structures that ultimately compress or irritate adjacent neural and vascular elements (3, 76, 77). For example, discogenic pain results from degeneration or disruption of the intervertebral disc (78–80). Facetogenic pain stems from osteoarthritic changes of zygapohyseal joints (facet) (81) and lumbar spinal stenosis is characterized by narrowing of the central canal or neural formina (82).

**Figure 3:**
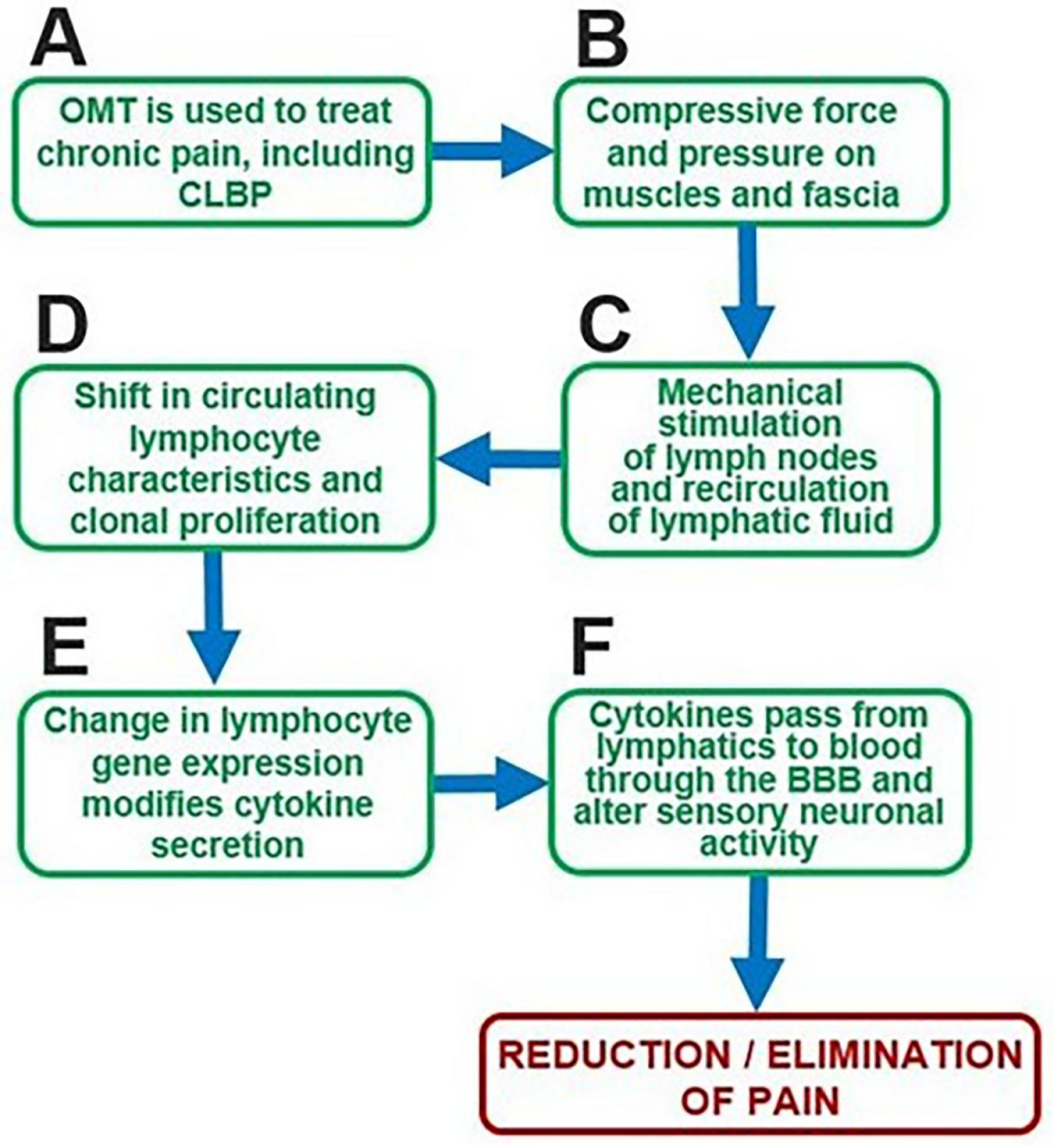
Proposed sequential model of pain reduction/elimination via OMT-induced immune modulation. **(A,B)** Steps involving the application of OMT. **(C-F)** Sequential steps demonstrating the immunomodulatory effects of OMT. BBB, blood-brain barrier.

The clinical effectiveness of OMT in managing pain is well supported by evidence (11–14). In patients with CLBP, meta-analyses and randomized controlled trials have consistently demonstrated significant reductions in pain intensity and improvements in functional status following OMT interventions (13–18). Compared to other non-pharmacologic approaches for CLBP, OMT offers a wider range of therapeutic benefits. Conventional physical therapy focuses on strengthening and mobility, and massage therapy targets soft tissue tension, but OMT integrates these elements with the added goal of restoring somatic and lymphatic function. By applying manipulative forces to muscles, fascia, and joints, OMT may influence both local biomechanics and systemic physiology, including circulation and immune activity. Whereas modalities such as physical therapy, chiropractic manipulation, massage therapy, and acupuncture primarily target localized dysfunction, OMT seeks to identify and treat somatic dysfunctions throughout the entire body to support the body's innate capacity for self-regulation and healing (14, 83). For example, in a patient presenting with low back pain, an osteopathic physician may identify dysfunctions in the sacrum, pelvis, ribs, cervical spine, or even cranial structures through a detailed palpatory examination, and then apply a range of hands-on techniques to restore structural balance and optimize function. This holistic approach may explain why OMT has shown greater improvements in pain and function relative to standard exercise or physiotherapy interventions. Together, these findings position OMT as a viable approach to managing chronic pain with the strong potential to reduce reliance on pharmacological therapies such as opioids and NSAIDs (Figure 3A).

### 2.4 OMT mechanically stimulates lymph nodes and impacts the lymphatic system

A recent review of CLBP pathophysiology highlights that persistent low back pian involves both peripheral inflammatory signaling and central sensitization mechanisms, including glial activation and immune-mediated nociceptive amplification (53). OMT for CLBP employs various techniques that apply direct and indirect forces to neuromusculoskeletal and vascular structures throughout the body. The forces applied create compression, relaxation, and distraction within these structures. These mechanical forces are detected by mechanosensitive cells within fascial and lymphatic tissues, including fibroblasts, macrophages and endothelial cells (65), which convert mechanical stimuli into biochemical signals through mechanotransduction pathways (84, 85). This process can alter cytokine release and immune cell activation, linking the mechanical effects of OMT to systemic physiological responses (85, 86). Some of OMT techniques are known as lymphatic pump techniques, however, many OMT techniques promote venous and lymphatic drainage through mechanical forces applied to the musculoskeletal tissues (87–91). Techniques like myofascial release involve the application of sustained pressure on muscles and soft tissue until tension is resolved (92). This type of manipulation also causes sustained mechanical stimulation of lymph nodes, altering lymphatic activity (86, 93). Another commonly used technique is muscle energy, which utilizes isometric contractions through a series of contract-relax cycles, to restore normal physiological motion, decrease muscle hypertonicity and improve circulatory and respiratory function (92). This technique can also activate local lymphatic pumps helping to redistribute lymphatic fluid. Furthermore, studies have demonstrated that muscle contraction and relaxation cycles can enhance the clearance of radiolabeled albumin within lymphatic channels (94).

Early osteopathic literature has emphasized the mechanical and autonomic regulation of lymphatic circulation, describing how fascial tension, diaphragmatic motion, and sympathetic tone can influence lymphatic contractility and fluid movement (95). Lymphatic pump techniques have been reported to enhance immune surveillance and leukocyte trafficking via manual augmentation of lymph flow under both normal and inflammatory conditions. In experimental models, increased thoracic duct lymph flow and mobilization of immune cells have been observed following lymphatic manipulation, supporting the concept that osteopathic techniques can influence immune cell distribution (89, 96).

Taken together, these findings support the concept that OMT techniques for CLBP may stimulate lymphatic activity and enhance lymph recirculation (Figures 3B,C).

### 2.5 OMT-Mediated impact on the lymphatic system leads to changes in proportions of circulating immune cells

Lymph nodes are innervated by sensory neurons that detect mechanical stimulation and modulate immune cell activity (97). OMT applies mechanical forces on lymph nodes and lymphatic vessels (98), resulting in significant changes in immune cell proportions and cytokine levels (89, 99–101). Alterations in leukocyte counts following OMT have been reported in both animal and human studies. In animal models, manipulation increased total leukocyte counts in thoracic and mesenteric duct lymph (89). These findings are supported by research in humans, including a randomized clinical trial in elderly adults, which reported a significant decrease in platelet counts following treatment (102). Additionally, a study in healthy adults reported reductions in CD117 + dendritic cells post-OMT (103). Collectively, these results suggest that the mechanical force exerted by OMT influences immune cell distribution via their effects on the lymphatic system (89, 98–102, 104). Moreover, OMT has been shown to modify cytokine levels (103–105). Mechanical strain patterns mimicking osteopathic techniques produce statistically significant reductions in pro-inflammatory mediators, including IL-6, IL-12, substance P, and TNF-α in a mouse model (106).

Clinical studies have similarly reported reductions in cytokines, including IL-8, granulocyte colony-stimulating factors, and TNF-α (103, 105). Notably, in a randomized controlled trial, researchers found that while several baseline cytokines correlated with somatic dysfunction severity, only TNF-α demonstrated a significant reduction after 12 weeks of OMT compared with sham treatment (105). Reductions in TNF-α were associated with improvements in pain and back-specific functioning, although other measured cytokines did not significantly change. These findings suggest that immune responses to OMT may be selective and highlight the complexity of cytokine involvement in CLBP.

These systemic changes suggest that local immune sensing may play a role in mediating OMT's downstream effects. For instance, tissue-resident macrophages can adapt to their physical surroundings, and changes in stiffness or stretch can influence their shape, motility, and activation through pathways involving integrins and the mechanosensitive channels Transient Receptor Potential Vanilloid 4 (TRPV4) and Piezo Type Mechanosensitive Ion Channel Component 1 (PIEZO1) (107). Within lymphatic and paraspinal tissues, these macrophages may help translate mechanical input from OMT into local immune signaling, bridging tissue-level forces with systemic inflammatory responses.

Taken together, these findings highlight that OMT-induced mechanical forces impact lymphatic function, altering immune-cell trafficking and cytokine signaling through changes in lymph flow and node activation, ultimately driving downstream changes in immune cell proportions and cytokine levels (Figure 3D).

### 2.6 Changes of immune system during the maintenance and resolution of pain

Immune system changes are crucial for the maintenance and resolution of pain, with cytokines and other inflammatory mediators playing a central role in this process. Cytokines produced by immune cells at local sites of inflammation can enter the bloodstream and cross the blood-brain barrier (BBB), where they interact with various cell types in the CNS (108). Three major pathways facilitate this interaction: the passage of circulating cytokines through leaky regions of the BBB, such as the circumventricular organs; active transport via specific cytokine transporters; and activation of cytokine receptors on afferent nerve fibers, which relay cytokine signals to the brain (109). Once in the CNS, cytokines can significantly alter cellular function. For example, cytokines injected directly into the brain stimulate astrocytes and microglia to produce additional cytokines, induce astrogliosis, and promote neovascularization (110–112). In addition, cytokines influence brain neurotransmission by increasing concentrations of certain biological amines, which are precursors for neurotransmitters such as serotonin and dopamine (113). Cytokines not only change the activity of various brain cells but can also activate signaling cascades which affect nociceptive signaling. Supporting this, intracerebroventricular injection of TNF, IL-1β, and IL-6 induces hyperalgesia (114). Conversely, blocking cytokine receptors or signaling pathways has been shown to reduce pain (115). Collectively, these findings highlight how modulation of cytokines and inflammatory mediators in the immune system can lead to changes in synaptic activity, pain modulation, and pain perception. The clinical relevance of these findings is underscored by the efficacy of immunotherapies targeting cytokines in treating painful inflammatory conditions. For instance, inhibitors of TNF-α and IL-1β have demonstrated success in alleviating pain associated with Crohn's disease, rheumatoid arthritis, and other chronic inflammatory disorders (116) (Figure 3F).

## 3 Discussion

### 3.1 Transcriptomic changes in lymphocytes serve as a bridge between OMT, immune system function, and pain perception

While current evidence demonstrates that OMT can shift immune-cell proportions and cytokine patterns (103–105), the link to specific transcriptomic changes remains unclear. Bridging this gap will require studies that can connect molecular findings with clinical outcomes. Future work should also explore whether the mechanical forces of OMT affect gene expression in tissue-resident immune cells, such as lymph-node macrophages, that may, in turn, influence the behavior of circulating immune cells.

Transcriptomic changes in circulating immune cells following OMT have been overlooked. Transcriptomic studies are important in elucidating important physiological mechanisms. From one side, recent advance in immune cell transcriptomics have underscored their utility in identifying genes and pathways associated with the development of CLBP (58, 59, 117). On the other side, recent animal studies demonstrated that OMT impacts transcriptomics in the neurons of rats (118). In a preclinical study, cranial osteopathic manipulation in aged rats significantly altered the expression of genes involved in neuronal signaling, particularly pathways linked to cholinergic activity and synaptic regulation. These transcriptomic changes were associated with improved spatial memory, suggesting that osteopathic mechanical forces can influence neural communication at the molecular level. While the study focused on brain tissue, it demonstrates how mechanical input can initiate gene-expression changes relevant to neuromodulation and supports extending this type of analysis to immune-cell populations (118).

Building on this foundation, we propose that transcriptomic analysis of circulating immune cells can reveal the mechanistic link between OMT, the immune system, and pain resolution. (Figure 3E). Specifically, we hypothesize that the mechanical stimulation provided by OMT influences lymphatic function, thereby modulating immune cell activity. This modulation is evidenced by changes in immune cell proportions and cytokine levels observed following treatment. These changes are likely driven by transcriptomic alterations that suppress the production of pro-inflammatory cytokines while promoting the secretion of anti-inflammatory cytokines. Consequently, OMT may mitigate transcriptomic changes in circulating immune cells (Figure 2) that perpetuate chronic pain, thereby contributing to pain alleviation.

Identifying these transcriptomic changes in immune cells following OMT provides a unique opportunity to explain the molecular mechanisms by which OMT alleviates chronic pain. This research holds a significant promise for informing the development of innovative strategies to address chronic pain conditions more effectively.

### 3.2 Hypothesis testing and future directions

Future investigations will require integrated clinical and molecular approaches to extensively evaluate the relationship between OMT, immune modulation, and pain outcomes in CLBP. This study would enroll adults with moderate to severe CLBP and sex-, age- and BMI-matched HCs. Participants would undergo several weekly sessions of standardized OMT, incorporating myofascial release, muscle energy techniques, and lymphatic maneuvers among other techniques. Blood samples would be collected at baseline, and after the final session. Pain and functional outcomes would be evaluated using validated survey tools at each session and three months post-treatment. The blood samples would be analyzed for cytokine levels (e.g., TNF-α, IL-1β, IL-6) across time points, and peripheral blood mononuclear cells (PBMCs) would be isolated for flow cytometry to identify shifts in immune cell proportions. Additionally, RNA sequencing in PBMCs would assess changes in transcriptomics. Correlations between changes in (a) cytokine levels, (b) transcriptomics, (c) lymphocyte subtype proportions, and (d) self-reported pain and functional outcomes will be assessed. To ensure specificity, a control arm involving sham OMT or equivalent manual therapies, such as massages or passive mobilization, should be included. Comparative transcriptomic profiling would help determine whether observed immune changes are unique to OMT's targeted biomechanical approach or common to general physical manipulation. Given the multifactorial nature of CLBP and immune regulation, validation of this hypothesis will likely require multi-center studies, longitudinal transcriptomic profiling, and mechanistic experiments in both human and preclinical models.

### 3.3 Implications

If validated, this hypothesis provides a compelling mechanistic framework for understanding the role of OMT in pain relief. By demonstrating that OMT modulates immune cell transcriptomics, this research could establish a more rigorous and defined biological mechanism for OMT efficacy, supporting its use as a non-pharmacological alternative to opioids and other medications with significant side effects. In addition, identifying transcriptomic signatures associated with OMT's therapeutic effects could facilitate the development of predictive objective biomarkers, reducing the reliance on subjective patient questionnaires. Beyond CLBP, these mechanisms may have broader implications, paving the way for innovative immunotherapies addressing inflammation and pain across a spectrum of immune-mediated conditions, including fibromyalgia, rheumatoid arthritis, and Long COVID.

## 4 Conclusion

In conclusion, this study is utilizing CLBP as a disease model and proposes a novel hypothesis describing how OMT alleviates pain through changes in the immune system. Though CLBP continues to be a leading cause of patient disability worldwide, the role of the immune system in its elimination continues to be overlooked. Evidence indicates that chronic pain can induce gene expression changes in circulating immune cells and that OMT has been associated with reductions in pain and modulation of lymphatic and immune parameters. However, researchers have yet to bridge the apparent gap in studying how the transcriptomic changes induced by OMT could be responsible for the reduction in pain. Thus, separate pieces of this process have been known, however, we have linked them to find the connection between OMT focused on the areas related to low back pain, the immune system and measured pain reduction. Our theory suggests that mechanical stimulation of soft tissue, and bony structures during OMT modifies immune cell gene expression, leading to reduced pro-inflammatory signaling, and reprogramming of maladaptive immune responses, ultimately resulting in pain reduction. Incorporating clinical and transcriptomic analyses into OMT treatment protocols will pave the way for future research aimed at identifying molecular biomarkers of OMT efficacy and elucidating its role in immune-mediated pain modulation. This integrative approach not only enhances our understanding of OMT's mechanisms but also establishes a foundation for the development of personalized, immune-targeted therapies for chronic pain.

## Data Availability

The original contributions presented in the study are included in the article/Supplementary Material, further inquiries can be directed to the corresponding authors.
